# Update on the Assessment of GFR in Patients with Cancer

**DOI:** 10.34067/KID.0000000736

**Published:** 2025-02-24

**Authors:** Verônica T. Costa e Silva, Fei Xiong, Lea Mantz, Meghan E. Sise, Sandra M. Herrmann, Abhijat Kitchlu

**Affiliations:** 1 Serviço de Nefrologia, Faculdade de Medicina, Instituto do Câncer do Estado de São Paulo, Universidade de São Paulo, São Paulo, Brazil; 2 Laboratório de Investigação Médica (LIM) 16, Faculdade de Medicina da Universidade de São Paulo, São Paulo, Brazil; 3 Division of Nephrology, Department of Medicine, University Health Network, University of Toronto, Toronto, Ontario, Canada; 4 Department of Radiology, Massachusetts General Hospital, Boston, Massachusetts; 5 Department of Diagnostic and Interventional Radiology, University Medical Center of the Johannes Gutenberg University Mainz, Mainz, Germany; 6 Division of Nephrology, Department of Medicine, Massachusetts General Hospital, Boston, Massachusetts; 7 Division of Nephrology and Hypertension, Mayo Clinic, Rochester, Minnesota

**Keywords:** cancer, chemotherapy, CKD, cisplatin, cisplatin nephrotoxicity, clinical trial, creatinine clearance, drug metabolism, renal function, onco-nephrology

## Abstract

Accurate assessment of GFR is key in patients with cancer to guide drug eligibility, adjust dosing of systemic therapy, and minimize the risks of undertreatment and systemic toxicity. Several aspects of GFR evaluation in patients with cancer have been unclear, such as the choice of the GFR estimating equation and the overall lack of data on the reliability of new filtration markers, such as cystatin C. This uncertainty has led to concerns that inaccurate GFR estimation may have a large effect on clinical practice and research. Recent data have brought important developments to the field. The new and timely Kidney Disease Improving Global Outcomes 2024 Clinical Practice Guideline for the Evaluation and Management of CKD raised important considerations and provided guidance on key aspects of GFR evaluation in patients with cancer. The guidelines cover valid estimating equations, incorporation of cystatin C in GFR estimation, drawbacks of using race in GFR estimation, and acknowledge that non-GFR determinants of filtration markers may be prominent in patients with cancer, reducing the accuracy of GFR estimating equations, prompting greater utilization of GFR measurement. The aim of this review is to summarize advances in GFR evaluation in patients with cancer considering the new Kidney Disease Improving Global Outcomes guidelines and other recent data.

## Introduction

Accurate assessment of GFR is crucial in the cancer setting to guide drug selection, adjust the dosing of systemic therapy, and minimize the risks of undertreatment and unnecessary kidney and systemic toxicities.^1^ Furthermore, decreased GFR remains a common exclusion criterion for randomized clinical trial of cancer therapies.^2^ However, significant developments in GFR evaluation in patients with cancer have occurred in the past few years. Recent studies have validated new GFR estimating equations compared with the underperformance of the long-used Cockcroft-Gault (CG),^3,4^ combined with a recent systematic review and position statement from the American Society of Onco-Nephrology (ASON) recommending the use of validated equations using both creatinine and cystatin C in this population.^5^

The Kidney Disease Improving Global Outcomes (KDIGO) 2024 Clinical Practice Guideline for the Evaluation and Management of CKD recognized patients with cancer as a population of interest, providing meaningful suggestions and recommendations.^6^ This review will address the intersection and translation into clinical practice of KDIGO's new recommendations in the context of cancer, including use of validated equations, incorporation of cystatin C in GFR estimation, the rationale for not including race in GFR estimation, and recognition of non-GFR determinants of filtration markers as particularly prominent in these patients. In addition, we provide an overview of body composition analysis as a tool to more accurately identify patients with sarcopenia, a brief summary on the clinical application of GFR measurement in patients with cancer, and review the history of carboplatin dosing as an example of a widely used chemotherapy that relies on accurate GFR determination.

## GFR Estimation and Considerations in Patients with Cancer

GFR estimation through equations involving endogenous filtration markers remains the most common practical approach to kidney function assessment in patients with cancer. Multiple GFR estimating equations have been developed,^5^ but until recently, there has been limited evidence or consensus recommendations to guide the selection of appropriate equations in patients with cancer. Three important recent developments may help guide contemporary GFR estimation in cancer: (*1*) an increase in the use of available validated equations to estimate GFR that are more accurate as compared with measured GFR (mGFR), (*2*) the availability of validated serum cystatin C-based and combined serum creatinine and cystatin C-based eGFR equations, and (*3*) studies assessing the performance of newer/updated validated equations (*e.g*., the 2021 CKD Epidemiology Collaboration [CKD-EPI] equation and the 2023 European Kidney Function Consortium [EKFC] equation).

### Choice of GFR Estimating Equations

The KDIGO 2024 Guidelines recommend using creatinine-based eGFR (eGFRcr) and that GFR estimation from the combination of creatinine and cystatin C (eGFRcr-cys) should be used in clinical situations when eGFRcr is less accurate and GFR affects clinical decision making.^6^ This is because eGFRcr-cys more closely reflects mGFR in the general population.^7,8^

**Figure 1:**
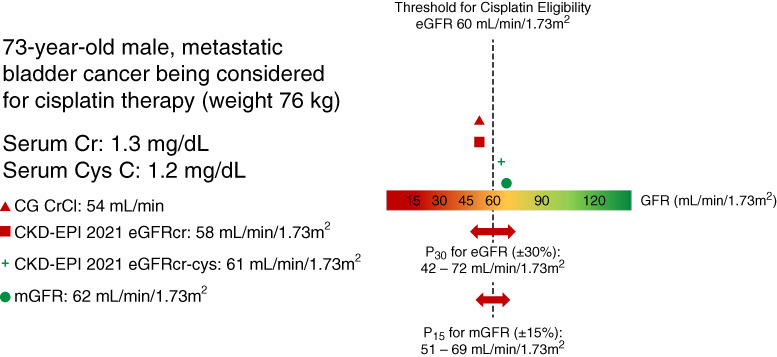
**Effect of GFR estimating equation and filtration markers on cisplatin eligibility.** Illustrative case of a patient under consideration for bladder cancer therapy with cisplatin. This patient's eligibility for cisplatin according to a commonly used threshold of 60 ml/min per 1.73 m^2^ may vary depending on the GFR estimating equation used and whether Cys C is incorporated into GFR assessment. Using CKD-EPI 2021 eGFRcr-cys and mGFR, the patient would be considered eligible, whereas through CG and CKD-EPI 2021 eGFRCr (without Cys C), the patient would be considered ineligible. CG, Cockcroft-Gault equation; CKD-EPI, CKD Epidemiology Collaboration equation; Cr, serum level of creatinine; CrCl, creatinine clearance; Cys C, serum level of cystatin C; eGFRcr, creatinine-based eGFR; eGFRcr-cys, GFR estimation from the combination of creatinine and cystatin C; mGFR, measured GFR; P_15_, percentage of estimates that differ up to 15% from the mGFR; P_30_, percentage of estimates that differ up to 30% from the mGFR.

The guidelines recommend that validated GFR estimating equations should be used, and specific criteria are provided to define this. Equations for GFR estimation should ideally be developed against mGFR by using exogenous filtration markers in a sufficient number of individuals with a wide range of clinical characteristics. Validated equations should be comparable and externally validated in separate populations with an acceptable threshold of precision and accuracy (Box 1). Bias (median difference of mGFR-eGFR) of <5, 5–10, and >10 ml/min per 1.73 m^2^ were considered small, medium, and large bias, respectively; 1−P_30_ (percentage of estimates that differed by more than 30% from mGFR) <10%, 10%–20%, and >20% were considered optimal, acceptable, and poor, respectively. Although these thresholds are suitable when comparing different equations to each other, the guidelines acknowledge that a variation of up to 30% may have clinical implications at an individual level. This is particularly relevant in scenarios where important clinical decisions need to be made, such as the eligibility and dosing of chemotherapy. Of note, there are no data assessing if the suggested thresholds of accuracy are related to adverse clinical events in patients with cancer. A hypothetical clinical example is shown in Figure 1, highlighting that mGFR is the preferred method for decision-making scenarios such as this. The KDIGO guidelines advised that filtration markers should be analyzed using standardized assays that are traceable to reference materials with a low coefficient of variation (under 2.3% and 2.0% for creatinine and cystatin C, respectively) and reported readily by clinical laboratories.^6^ An enzymatic method to assay creatinine is suggested, where possible, and cystatin C and creatinine should be measured in the same sample to enable calculation of eGFRcr-cys.

Box 1. **Commonly used performance metrics for GFR estimating equations**

- Accuracy: defined as the percentage of estimates that differed by more than 30% from the measured GFR (mGFR; 1−P_30_) and represents the percentage of large errors, which can be clinically significant. A 1−P_30_ of 10%–20% is generally considered adequate for many clinical decisions.
- Bias: defined as the median of the differences between mGFR and eGFR and provides insight into non-GFR determinants of the filtration markers. Positive and negative values represent and underestimate and overestimate of mGFR, respectively.
- Precision: is expressed as the interquartile range of the differences between mGFR and eGFR.

Importantly, older equations that remain in widespread clinical use for decisions related to cancer therapy eligibility and modification (*e.g*., CG and Modification of Diet in Renal Disease, among others) would not meet these criteria and therefore would not be considered validated per KDIGO. In addition, the CG equation was derived against measured 24-hour urine-creatinine clearance (and therefore provides an estimated creatinine clearance rather than GFR), uses an empiric (expert opinion-based) correction factor for sex, and likely substantially overestimates muscle mass and, therefore, GFR in the setting of obesity and sarcopenia.^9^

Of note, the KDIGO guidelines recommend the use of body surface area–adjusted eGFR values (*e.g*., adjusted according to patient's actual body surface area) for drug dosing consideration in patients with extreme obesity in the general population, and this is likely to be applicable in patients with cancer.^6^ Excluding obesity and extremes of weight, for most anticancer drugs, standardized eGFR are usually advised for dose adjustment, except for carboplatin.^1^

## Non-GFR Determinants of Filtration Markers in Patients with Cancer

In the KDIGO guidelines, patients with cancer are noted as a population in which non-GFR determinants of both creatinine and cystatin C may be more profound, requiring a better understanding of these elements.

### Non-GFR Determinants of Serum Creatinine

Creatinine is derived by the metabolism of phosphocreatine in muscle, from dietary protein intake, and removed from the body through glomerular filtration and proximal tubular secretion (such as 10%–40% of filtered load) *via* organic cation transporter 2 and multidrug and toxin extrusion 1. Thus, apart from GFR, creatinine is affected by muscle mass, diet, age, sex, race, and drugs that influence tubular secretion such as trimethoprim, cimetidine, and multiple tyrosine kinase and CDK4/6 inhibitors used in the treatment of various cancers.^5,10^

In patients with cancer, the most critical non-GFR determinants of creatinine are changes in muscle mass and food ingestion. Sarcopenia occurs in around 40%–50% of patients,^11^ varying from 16% of those before cancer treatment^12^ to up to 70% of late-stage patients undergoing chemotherapy.^13^ Conversely, overweight/obesity can be observed in up to 60% of patients with cancer, particularly in certain cancer sites (breast, prostate, and thyroid).^14,15^ Of note, reduced food ingestion is frequently observed due to cancer itself, particularly in the case of tumors from the gastrointestinal site or complications from cancer treatment, such as large surgeries or chemotherapy, and can affect the metabolism of serum creatinine even before weight change and clinical signs of sarcopenia are present.^16^ Patients with reduced total dietary protein intake have been reported to have significantly lower serum creatinine values and therefore higher serum eGFRcr values.^17^ This aspect can be easily overlooked, particularly in overweight/obese patients, who may seem fit, but are actually sarcopenic,^18^ and should be considered when assessing non-GFR determinants of serum creatinine.

### Non-GFR Determinants of Serum Cystatin C

Cystatin C is a naturally occurring inhibitor of lysosomal cysteine proteinases, expressed in all nucleated cells. Approximately 99% of the filtered load of cystatin C is reabsorbed by the proximal tubular cells, where it is almost completely catabolized, with the remainder eliminated largely intact in the urine.^8^ Cystatin C is not influenced by muscle mass or diet and, compared with serum creatinine, is less affected by age, sex, race, and world region, thus suitable for sarcopenic patients. However, cystatin C is affected by smoking, inflammation, high adiposity, thyroid function, and use of glucocorticoids.^19^ Of note, the non-GFR determinants of cystatin C are less clearly understood than those of creatinine.^8^

In malignancy, cystatin C is extensively expressed in cells and tissues of various solid tumors. Some clinical studies demonstrate increased levels of serum cystatin C in patients with active cancer, namely, breast, head and neck, lung, colorectal, prostate, and kidney cancers.^20^ However, these studies lacked adjustment for mGFR and assessment of other non-GFR determinants. Of note, most studies evaluating the non-GFR determinants of cystatin C did not include patients with cancer,^19,21^ and the incidence of factors such as thyroid dysfunction and corticosteroid exposure in patients with cancer is not well known and likely varies throughout cancer treatment.

## Clinical Studies Supporting Cystatin C Inclusion in GFR Estimation in Patients with Cancer

Studies have sought to assess the performance of various GFR equations in patients with cancer, recently including those incorporating both serum creatinine and cystatin C. The ASON Position Statement Committee conducted a systematic review to assess the accuracy of GFR estimating equations versus mGFR as determined through urinary or plasma clearance of an exogenous filtration marker.^5^ A total of 39 studies were included, and the total number of patients was 21,949 (19,025 with solid cancers, 1146 with hematologic cancers, and 1778 with unspecified malignancies). Versions of the CKD-EPI equation were most frequently concluded as the most accurate GFR estimating equation (38% or 13 of 34 studies comparing multiple eGFR formulas).

Importantly, all studies that assessed versions of CKD-EPI on the basis of creatinine and cystatin C (CKD-EPIcr-cys) reported P_30_ values >80%. The largest included cohort, the Onco-GFR study by Costa e Silva *et al.*, included 1200 participants with solid tumors and demonstrated that CKD-EPIcr-cys (2012) had the best performance: bias −2.0 (−2.6 to −1.1) ml/min per 1.73 m^2^ and 1−P_30_ 7.8 (6.3–9.4)%. In this analysis, CG was the least accurate, 1−P_30_ was 24.9 (22.4 to 27.3)%, and mean bias was –8.1 (–9.4 to –6.7) ml/min per 1.73 m^2^.^4^ Notably, after adjustment for demographic and clinical variables, a strong association between different cancer sites and cystatin C independent of mGFR (largest effect <6%) was not observed. In addition, non-GFR determinants of cystatin C were different compared with those affecting serum creatinine, but did not seem to have a greater influence on GFR estimation.

**Figure 2:**
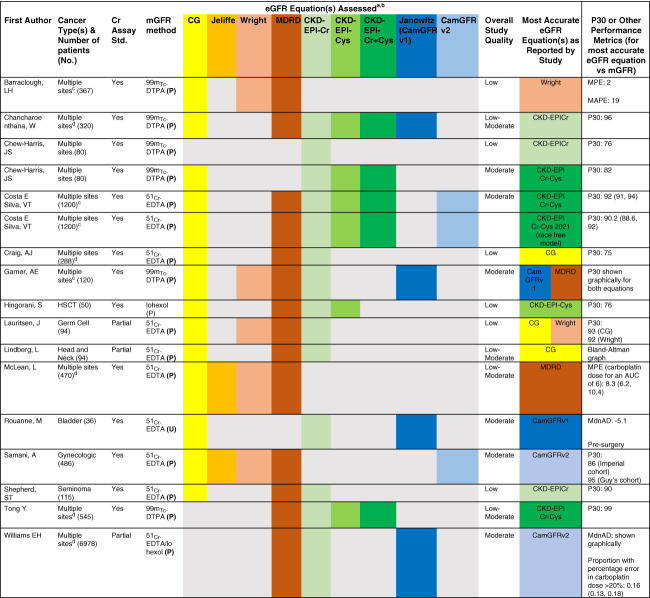
**Summary of studies of the systematic review assessing GFR estimating equations in patients with cancer.** Reproduced with permission from ref. 5. ^a^Studies reporting use of standardized serum creatinine assay and GFR measurement on the basis of plasma or urinary clearance are included in the above table. ^b^Color-coding reflects GFR estimating equation inclusion for assessment within each study: CG (yellow), Jeliffe (orange); Wright (light brown); MDRD equation (brown); CKD-EPI Cr (light green); Cys C (medium green); Cr-Cys (dark green); Janowitz/CamGFRv1 (dark blue); CamGFRv2 (light blue). ^c^Solid tumors. ^d^Mix of solid tumors and hematology cancers. For studies reporting P30, other reported metrics were omitted. For studies not reporting P30, we focused on mean or median percentage errors and mean or median absolute percentage errors. Mean percentage errors were calculated from the proportional differences between eGFR and mGFR expressed as %. Negative and positive values reflect over and underestimate of the mGFR, respectively. MAPE was calculated from the proportional differences between eGFR and mGFR expressed as % but does not incorporate positive or negative signs. MdnAD calculated from the subtraction of eGFR-mGFR expressed as ml/min per 1.73 m^2^. Variation of reported metrics was described when available in the original paper as follows: 95% CI (separated by comma), interquartile range (separated by hyphen), and SD (within parenthesis). P30 is the percentage of estimates that differed by more than 30% from the measured GFR. AUC, area under the curve; CamGFRv1, Cambridge GFR version 1; CamGFRv2, Cambridge GFR version 2; CI, confidence interval; Cr-Cys, creatinine and Cys C; EDTA, ethylenediaminetetraacetic acid; DTPA, diethylenetriaminepentaacetic acid; HSCT, hematopoietic stem cell transplantation; MAPE, mean absolute percentage error; MdnAD, median absolute difference; MDRD, Modification of Diet in Renal Disease; MPE, mean percentage error; P, plasmatic clearance; U, urinary clearance; UN, unknown.

Overall, on the basis of the available data for validated equations for eGFRcr-cys (involving 2508 patients in whom CKD-EPIcr-cys was assessed in eight studies; Figure 2), the ASON Position Statement Committee advocated for the use of validated equation incorporating both serum creatinine and cystatin C.

After this position statement, a recent analysis by Fu *et al.* assessed the performance of multiple eGFR equations, including the EKFC equations, in a large cross-sectional cohort in Sweden, including a subgroup of 2668 patients with cancer.^22^ In this subgroup, both EKFC and 2021 CKD-EPI using both creatinine and cystatin C had similar performance, with P_30_ 92.1 (91.1 to 93.2)% and 90.6 (89.4 to 91.8)%, respectively. These findings support the use of widely available, validated eGFR equations that combine creatinine and cystatin C in patients with cancer, with avoidance of older, nonvalidated equations, including CG for clinical decision making.

## Body Composition and GFR Estimation

Low muscle mass is the most important non-GFR determinant of serum creatinine in patients with cancer, whereas high adiposity significantly affects cystatin C.^21^ In the Onco-GFR study, the reported bias for CKD-EPI on the basis of creatinine (CKD-EPIcr), cystatin C (CKD-EPIcys), and CKD-EPIcr-cys for patients with body mass index (BMI) <20 kg/m^2^ were −16.8 (−21.4 to −13.6), −1.4 (−4.8 to 3.9), and −8.9 (−14.2 to −6.1) ml/min per 1.73 m^2^, respectively. In the case of patients with BMI >30 kg/m^2^, bias for those equations was −4.5 (−6.0 to −2.5), 6.9 (5.9 to 8.4), and 1.1 (−0.2 to 2.4) ml/min per 1.73 m^2^, respectively.^4^ This result suggests that individual patients' body composition affects GFR estimation. In this scenario, an accurate body composition assessment is crucial to identify patients prone to having more significant bias and worse accuracy in GFR estimation, in which mGFR would be preferred for decision making. Owing to the rise in obesity, identifying visibly wasted patients is increasingly rare in clinical practice, and clinicians must look beyond weight and BMI as sole indicators of muscle loss. Skeletal muscle mass can be determined using dual-energy X-ray absorptiometry, magnetic resonance imaging, or computed tomography (CT) scans.^23,24^ This is particularly relevant given the increasing incidence of sarcopenic obesity, defined by underlying depleted muscle mass in patients with elevated BMI.^18^ Sarcopenic obesity is an increasingly common clinical entity and is associated with an increased risk of adverse events and mortality in patients with cancer.^18,25–27^

**Figure 3:**
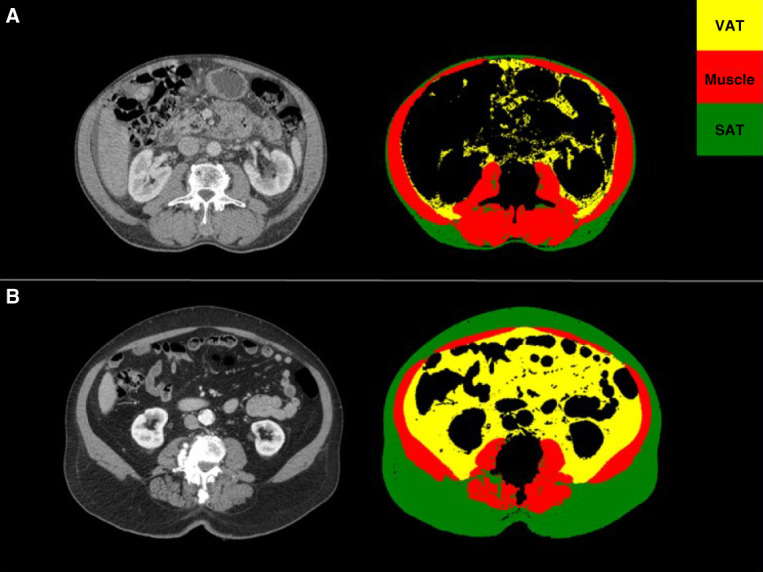
**Measured and eGFR in two patients with body composition parameters assessed by CT scan.** Skeletal muscle area is shown in red, VAT in yellow, and SAT in green. (A) A 68-year-old man with a normal BMI (23.8 kg/m^2^) and normal SMI (64.5 cm^2^/m^2^). Serum creatinine was 1.1 mg/dl; serum Cys C was 1.2 mg/L. His mGFR was 67 ml/min per 1.73 m^2^; eGFRcr was 73 ml/min per 1.73 m^2^; eGFRcys was 59 ml/min per 1.73 m^2^; eGFRcr-cys was 68 ml/min per 1.73 m^2^. (B) A 77-year-old man with a high BMI (39.9 kg/m^2^) and normal SMI (66.7 cm^2^/m^2^). Serum creatinine was 1.3 mg/dl; serum Cys C was 1.4 mg/L. His mGFR was 61 ml/min per 1.73 m^2^; eGFRcr was 57 ml/min per 1.73 m^2^; eGFRcys was 46 ml/min per 1.73 m^2^; eGFRcr-cys was 53 ml/min per 1.73 m^2^. GFR was estimated using the 2021 CKD-EPI equations. Sarcopenia is defined by a SMI <55 cm^2^/m^2^ for men. BMI, body mass index; CT, computed tomography; eGFRcys, eGFR based on the serum level of cystatin C; SAT, subcutaneous adiposity tissue; SMI, skeletal muscle index; VAT, visceral adiposity tissue.

Recently, Hanna *et al.* evaluated the relationship between body composition defined by CT scans and discordance between eGFRcr and eGFR on the basis of the serum level of cystatin C in 545 adult patients with cancer.^28^ Muscle and adipose tissue cross-sectional areas were measured at the third lumbar vertebral body level using a validated deep learning pipeline. In total, 320 (58.7%) patients met the criteria for CT-defined sarcopenia, and 136 (25%) had high adiposity. Of note, 60 (44.1%) patients with high adiposity were sarcopenic. After adjustment for potential confounders, CT-defined sarcopenia and high adiposity were both associated with *>*30% eGFR discordance (eGFR on the basis of the serum level of cystatin C-eGFRcr): odds ratio, 1.90 (1.12–3.24); odds ratio, 2.01 (1.15–3.52), respectively. The authors conclude that both sarcopenia and high adiposity were independently associated with large eGFR discordance, and CT scans can help interpret GFR estimates. Although this study lacks mGFR and the true accuracy of eGFR equations could not be determined, this result supports CT scans as a promising instrument to identify patients more likely to have worse accuracy in eGFR. In a subsequent analysis of the same cohort, the authors concluded that patients with discordant eGFR estimates were at higher risk of medication-related adverse events when receiving renally cleared medications.^29^ This highlights the fact that harm could be occurring in patients with inaccurate GFR estimates and the need to generate more personalized strategies to estimate GFR. CT scans are readily available and performed frequently as a part of routine cancer surveillance. Although body composition parameters are not routinely available, there are validated image analysis pipelines increasingly used which allow for automated body composition analysis.^30–33^ Thus, in the near future, it may be possible to incorporate body composition information from CT scans into clinical care. For illustration, body composition parameters, combined with mGFR and eGFR of two patients (one obese nonsarcopenic and one within normal limits of muscle and fat mass), are depicted in Figure 3.

## Effect of Race on GFR Estimation

The KDIGO guidelines emphasize that race should not be used in the GFR estimating equations,^6^ acknowledging that race is dynamic, influenced by cultural, geographic, and sociopolitical aspects, which can change across geography and over time.^34^

There are limited data assessing the effect of including/removing race in GFR estimation in patients with cancer. Because the race-free 2021 CKD-EPIcr equation has been shown to lead to lower eGFR values in Black patients,^35^ there is a concern that Black patients could be at risk of having their chemotherapy underdosed or be less likely to meet GFR cutoffs for clinical trials eligibility. Two recent studies tried to assess this issue.^36,37^ Casal *et al.* conducted a retrospective study including 340 Black patients enrolled in phase 1 randomized clinical trials between January 1995 and October 2010 who had eGFRcr calculated using the 2009 CKD-EPI equation and its version without the race term.^36^ Dosing simulations were performed for ten anticancer drugs with GFR cutoffs for dosing or eligibility. It was demonstrated that the number of patients ineligible for therapy or recommended to receive any renal dose adjustment increased between 61% and 163% depending on the drug when the 2009 CKD-EPI with the race term was used compared with the CKD-EPI 2009 equation with race, with up to 18% of patients with discordant recommendations. The authors conclude that removing race from the CKD-EPI equation could lead to undertreatment of Black patients with cancer and adversely affect their outcomes. The same research group used the same methodology and dataset to compare the odds of cisplatin ineligibility using 2021 CKD-EPI versus 2009 CKD-EPI (with the term for race) equations.^37^ They demonstrated that Black patients had 48% higher odds of cisplatin ineligibility and non-Black patients had 27% lower odds of ineligibility using 2021 CKD-EPI (*P* < 0.001). However, those studies did not have mGFR as a comparator, so it is unclear whether dose adjustment and reduced eligibility would be correctly or inappropriately prescribed in real life if decisions were made using race-free versions of the assessed equations compared with the models which included race adjustment. In addition, cystatin C was not available to assess if race disparity would be attenuated if eGFRcr-cys had been used. Until more data are available, it is reasonable to consider directly measuring GFR or incorporating cystatin C for Black patients with eGFR values close to the threshold for dosing and/or eligibility for certain drugs.

A subsequent analysis of the Onco-GFR Study assessed the 2021 CKD-EPI race-free equations. Bias of CKD-EPIcr and CKD-EPIcr-cys was −10.0 (−10.7 to −8.8) and −4.1 (−4.8 to −3.3) ml/min per 1.73 m^2^, respectively; 1−P_30_ was 22.3 (19.8–24.4)% and 9.8 (8.0–11.4)%.^38^ The CKD-EPI race-free equations were slightly less accurate compared with the models with race. However, the difference was not substantial, and incorporating cystatin C into the eGFRcr race-free equation reduced racial differences, supporting their suitability for treatment decision making. The EKFC models were assessed in the same cohort. It was observed that versions including serum creatinine (1−P_30_: 10.0 [8.3%–11.7%]) and combining creatinine and cystatin C (1−P_30_: 5.3 [4.1%–6.4%]) presented optimal performance.^39^ Although this study did not assess the effect of removing race on drug dosing or eligibility, these results support the KDIGO recommendations of removing race from GFR estimation in patients with cancer.

## GFR Measurement

The KDIGO guidelines acknowledge that mGFR is particularly useful in patients with cancer and describe that GFR measurement is indicated for drug dosing of chemotherapies cleared by the kidney, namely, but not restricted to carboplatin, cisplatin, and methotrexate.^6^ Currently, the plasma clearance of iohexol and chromium 51-labeled EDTA is the most commonly used methodologies in clinical practice nowadays, with iohexol increasingly used in recent years, particularly in European countries.^40^ The EKFC together with the European Federation of Clinical Chemistry and Laboratory Medicine recently published recommendations for GFR measurement using 1-compartment protocols for iohexol plasma clearance to provide standardized procedures.^41^ GFR is calculated using a kinetic slope-intercept model, including 2–6 plasma samples with an initial sample obtained at 2 hours after injection and the timing of the final sample ranging from 4 to 24 hours, depending on expected GFR. Late collections (8–24 hours) are advised if the eGFR is below 30 ml/min per 1.73 m^2^. This same rationale and procedure methodology may apply for the plasma clearance of filtration markers such as chromium 51-labeled EDTA.^4,42^ We agree with the recommendation of choosing the multiple samples protocol instead of the single sample procedure for decisions on chemotherapy dosing and eligibility in patients with cancer.

## Effect of GFR Evaluation on Carboplatin Dosing

**Figure 4:**
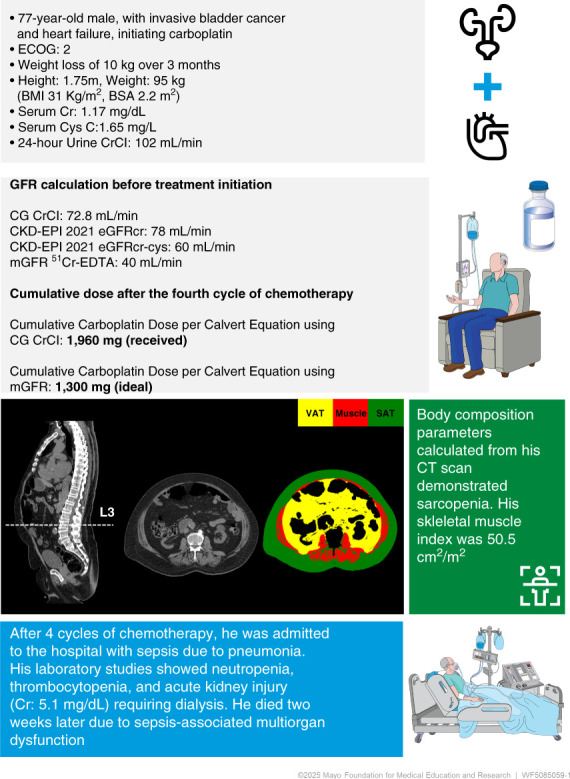
**Illustrative case of carboplatin dosing in a patient with bladder cancer.** This patient initiating carboplatin treatment has significant sarcopenia, resulting in overestimation of GFR through CG versus his mGFR. He received carboplatin dose as per the Calvert equation incorporating CG at a considerably higher dose (46% greater) than what would be calculated using mGFR and subsequently experienced poor kidney and systemic adverse events. Sarcopenia is defined by a SMI <55 cm^2^/m^2^ for men. BSA, body surface area; CKD-EPIcr, CKD-EPI based on Cr; CKD-EPIcr-cys, CKD-EPI based on Cr and Cys C; ECOG, Easter Cooperative Oncology Group; mGFR ^51^Cr-EDTA, mGFR using plasma clearance of chromium-51 labeled EDTA. Sarcopenia is defined by a skeletal muscle index < 55 cm^2^/m^2^ for men.

The KDIGO guidelines state that mGFR may be the preferred method for guiding initial carboplatin dosing.^6^ This is because carboplatin is primarily eliminated and is associated with a unique challenge of accurate GFR evaluation to be inputted in the Calvert equation for dose determination, which has changed over time (Supplemental Material). Recently, the International Consensus Guideline for Anticancer Drug Dosing in Kidney Disease provided recommendations on carboplatin dosing, including using the Calvert equation on the basis of mGFR as the preferred methodology, particularly in the case of curative intent, extremes of body composition, and skeletal muscle waste conditions (Table 1).^43^ To illustrate the challenges in carboplatin prescription, we describe a real case in which an elderly obese sarcopenic patient with bladder cancer received a carboplatin dose calculated through the CG 46.1% higher compared with the ideal dose provided by the mGFR performed (Figure 4).

**Table 1:** Carboplatin dose recommendations according to kidney function^a^

Intravenous Carboplatin Dosing Recommendation^b^		
eGFR (ml/min per 1.73 m^2^)	Dose	Comment
≥60	Target AUC using Calvert formula^c^^,^^d^	Directly mGFR^e^ is the preferred kidney function value in the Calvert formula, especially when *either* • Treatment intent is curative • Patient has extremes of body composition, conditions of skeletal muscle, is an amputee or is paraplegic • eGFR >125 ml/min per 1.73 m^2^ If estimating kidney function, BSA-adjusted eGFR^f^ is preferred as the kidney function value in the Calvert formula Capping of kidney function is not recommended^g^
45–59	Target AUC using Calvert formula^c^^,^^d^	Directly mGFR^e^ is the preferred kidney function value in the Calvert formula especially when either: • Treatment intent is curative • Patient has extremes of body composition, conditions of skeletal muscle, is an amputee or is paraplegic If estimating kidney function, BSA-adjusted eGFR^f^ is preferred as the kidney function value in the Calvert formula Increased risk of adverse events (*i.e*., thrombocytopenia, leukopenia), especially in patients with either a poor performance status, extensive previous anticancer treatment, or concomitant nephrotoxic drug exposure
30–44	Target AUC using Calvert formula^c^^,^^d^	Directly mGFR^e^ is the preferred kidney function value in the Calvert formula Increased risk of adverse events (*i.e*., thrombocytopenia, leukopenia), especially in patients with either a poor performance Status, extensive previous anticancer treatment, or concomitant nephrotoxic drug exposure
15–29		
<15 (without KRT)	Consult a multidisciplinary team consisting of oncology/hematology with nephrology and/or clinical pharmacology for the management of dosing	
KRT		

DuBois equations to calculate body surface area. Online calculator available at: https://www.eviq.org.au/p/4171. AUC, area under the concentration-time curve; BSA, body surface area; CKD-EPI, CKD Epidemiology Collaboration; mGFR, measured GFR.

a Reproduced with permission from refs. 35 and 43.

b For bone marrow transplantation conditioning protocols, consult the transplant team if the patient has kidney dysfunction and is requiring carboplatin as part of their treatment. The dose adjustments have not been tailored for these protocols

c Recalculation of carboplatin doses at each cycle is unnecessary, except when baseline kidney function (*e.g*., eGFR) alters by >20% or when there is a change in the clinical status of the patient.

d Calvert formula: dose (mg)=target area under the curve (mg ml^−1^ min)×(GFR [ml/min]+25 [ml/min]).

e Measured GFR refers to a direct measurement of the clearance of exogenous markers such as iohexol, iothalamate, chromium-51 labeled EDTA or technetium-diethylenetriaminepentaacetic acid.

f Body surface area–adjusted eGFR (ml/min) *via* the CKD Epidemiology Collaboration equation=(eGFR [ml/min per 1.73 m^2^]×body surface area [m^2^])/1.73. Use either Mosteller or DuBois

g Capping kidney function to 125 ml/min per 1.73 m^2^ for use in the Calvert formula may reduce therapeutic efficacy without reducing toxicity. When automated laboratory eGFR values are reported as greater than an upper limit (*e.g*., eGFR ≥90 ml/min per 1.73 m^2^), manual calculation of eGFR *via* the CKD Epidemiology Collaboration equation is required before applying this value to the body surface area-adjusted eGFR in the Calvert formula.

## Future Perspectives on GFR Evaluation in Patients with Cancer

Two new methodologies are being developed for direct and simplified quantitative GFR measurement: one technique uses a novel fluorescein carboxy-methylated dextran (rapidly filtered by the kidney) and the other uses a transdermal sensor to measure the removal of a fluorescent tracer (relmapirazin) from the blood.^44^ Recently, in a phase 2 observational study, the plasma clearance of relmapirazin was compared with that of iohexol in 120 normal individuals and patients with CKD with eGFRcr of 15–120 ml/min per 1.73 m^2^.^45^ Bland-Altman analysis demonstrated a difference of −0.7 (5.6 to −7) ml/min per 1.73 m^2^, and P_30_ was 100 (96.5–100)%. Despite the accuracy of transdermal detection of relmapirazin for mGFR is to be determined and the lack of data on patients with cancer, these techniques are promising, may refine drug dosing in clinical practice, and could be used to develop more precise dosing guidelines during drug development.

In conclusion, best practices for GFR evaluation in patients with cancer are evolving quickly. We encourage nephrologists, oncologists, and cancer caregivers to embrace the new KDIGO recommendations since they represent an essential step forward in improving GFR evaluation in patients with cancer, providing guidance for clinical practice and research, and improving the kidney care of patients with cancer.

## Supplementary Material

SUPPLEMENTARY MATERIAL

## Supplemental Material

Supplemental Material. Carboplatin dosing according to GFR.

Supplemental References
