# Supplementary material for: Update on the Assessment of GFR in Patients with Cancer
Source: Kidney360. 2025 Feb 24;6(5):861–70. doi: 10.34067/KID.0000000736 (PMC12136654; doi:10.34067/KID.0000000736)
Supplement: SUPPLEMENTARY MATERIAL [file kidney360-6-861-s001.pdf]

ASN American Society of Nephrology
JASN Journal of the American Society of Nephrology
KIDNEY360 Accessing Our World From Every Angle
CJASN Clinical Journal of the American Society of Nephrology

ASN Journal Disclosure Form

As per ASN journal policy, I have disclosed any financial relationships or commitments I have held in the past 36 months as included below. I have listed my Current Employer below to indicate there is a relationship requiring disclosure. If no relationship exists, my Current Employer is not listed.

V. Costa e Silva reports the following:
Other Interests or Relationships: I am an associate editor of the PlosOne journal and coordinator of the GFR research group of the American Society of Onco-Nephrology

I understand that the information above will be published within the journal article, if accepted, and that failure to comply and/or to accurately and completely report the potential financial conflicts of interest could lead to the following: 1) Prior to publication, article rejection, or 2) Post-publication, sanctions ranging from, but not limited to, issuing a correction, reporting the inaccurate information to the authors' institution, banning authors from submitting work to ASN journals for varying lengths of time, and/or retraction of the published work.

Name: Veronica Torres Costa e Silva
Manuscript ID: K360-2024-000812R1
Manuscript Title: Update on the Assessment of Glomerular Filtration Rate in Patients with Cancer
Date of Completion: January 30, 2025
Disclosure Updated Date: January 30, 2025

ASN
American Society of Nephrology
JASN
Journal of the American Society of Nephrology
KIDNEY360
Accessing Our World From Every Angle
CJASN
Clinical Journal of the American Society of Nephrology

ASN Journal Disclosure Form

As per ASN journal policy, I have disclosed any financial relationships or commitments I have held in the past 36 months as included below. I have listed my Current Employer below to indicate there is a relationship requiring disclosure. If no relationship exists, my Current Employer is not listed.

S. Herrmann reports the following:
Employer: Mayo Clinic; Patents or Royalties: Pfizer,but this is not related to the current research; and Advisory or Leadership Role: Associate editor Journal of Onconephrology and Nephrology Dialysis and Transplantation Journal; Founding member of American Society of Onconephrology.

I understand that the information above will be published within the journal article, if accepted, and that failure to comply and/or to accurately and completely report the potential financial conflicts of interest could lead to the following: 1) Prior to publication, article rejection, or 2) Post-publication, sanctions ranging from, but not limited to, issuing a correction, reporting the inaccurate information to the authors' institution, banning authors from submitting work to ASN journals for varying lengths of time, and/or retraction of the published work.

Name: Sandra Herrmann
Manuscript ID: K360-2024-000812R1
Manuscript Title: Update on the Assessment of Glomerular Filtration Rate in Patients with Cancer
Date of Completion: February 3, 2025
Disclosure Updated Date: April 10, 2024

ASN
American Society of Nephrology
JASN
Journal of the American Society of Nephrology
KIDNEY360
Accessing Our World From Every Angle
CJASN
Clinical Journal of the American Society of Nephrology

ASN Journal Disclosure Form

As per ASN journal policy, I have disclosed any financial relationships or commitments I have held in the past 36 months as included below. I have listed my Current Employer below to indicate there is a relationship requiring disclosure. If no relationship exists, my Current Employer is not listed.

A. Kitchlu reports the following:
Employer: University of Toronto; and Other Interests or Relationships: Amgen, Inc., Jassen, Inc. (University Health Network Onco-Nephrology Fellowship Program Funding).

I understand that the information above will be published within the journal article, if accepted, and that failure to comply and/or to accurately and completely report the potential financial conflicts of interest could lead to the following: 1) Prior to publication, article rejection, or 2) Post-publication, sanctions ranging from, but not limited to, issuing a correction, reporting the inaccurate information to the authors' institution, banning authors from submitting work to ASN journals for varying lengths of time, and/or retraction of the published work.

Name: Abhijat Kitchlu
Manuscript ID: K360-2024-000812R1
Manuscript Title: pdate on the Assessment of Glomerular Filtration Rate in Patients with Cancer
Date of Completion: February 6, 2025
Disclosure Updated Date: April 16, 2024

ASN American Society of Nephrology
JASN Journal of the American Society of Nephrology
KIDNEY360 Accessing Our World From Every Angle
CJASN Clinical Journal of the American Society of Nephrology

ASN Journal Disclosure Form

As per ASN journal policy, I have disclosed any financial relationships or commitments I have held in the past 36 months as included below. I have listed my Current Employer below to indicate there is a relationship requiring disclosure. If no relationship exists, my Current Employer is not listed.

L. Mantz reports the following:
Employer: Massachusetts General Hospital

I understand that the information above will be published within the journal article, if accepted, and that failure to comply and/or to accurately and completely report the potential financial conflicts of interest could lead to the following: 1) Prior to publication, article rejection, or 2) Post-publication, sanctions ranging from, but not limited to, issuing a correction, reporting the inaccurate information to the authors' institution, banning authors from submitting work to ASN journals for varying lengths of time, and/or retraction of the published work.

Name: Lea Mantz
Manuscript ID: K360-2024-000812R1
Manuscript Title: Update on the Assessment of Glomerular Filtration Rate in Patients with Cancer.
Date of Completion: January 19, 2025
Disclosure Updated Date: January 19, 2025

ASN American Society of Nephrology · JASN Journal of the American Society of Nephrology · KIDNEY360 Accessing Our World From Every Angle · CJASN Clinical Journal of the American Society of Nephrology

ASN Journal Disclosure Form

As per ASN journal policy, I have disclosed any financial relationships or commitments I have held in the past 36 months as included below. I have listed my Current Employer below to indicate there is a relationship requiring disclosure. If no relationship exists, my Current Employer is not listed.

M. Sise reports the following:
Employer: Massachusetts General Hospital; Consultancy: Vera, Travere, Calliditas, Mallinckrodt, Novartis, Otsuka, Alpine Immune sciences/Vertex, RelayTx, Medibeacon, Merida biosciences (self). Emed, X-Biotix (Spouse); Ownership Interest: X-Biotix (spouse); Research Funding: Angion, Otsuka, Gilead, Cabaletta, Novartis, Roche/Genetech, Merck; Patents or Royalties: US 11,441,196 B2 : Roby Bhattacharyya; RIBOSOMAL RIBONUCLEIC ACID HYBRIDIZATION FOR ORGANISM IDENTIFICATION; and Advisory or Leadership Role: X-Biotix (spouse).

I understand that the information above will be published within the journal article, if accepted, and that failure to comply and/or to accurately and completely report the potential financial conflicts of interest could lead to the following: 1) Prior to publication, article rejection, or 2) Post-publication, sanctions ranging from, but not limited to, issuing a correction, reporting the inaccurate information to the authors' institution, banning authors from submitting work to ASN journals for varying lengths of time, and/or retraction of the published work.

Name: Meghan E. Sise
Manuscript ID: K360-2024-000812R1
Manuscript Title: K360-2024-000812R1) and ManuscriUpdate on the Assessment of Glomerular Filtration Rate in Patients with Cancer
Date of Completion: January 31, 2025
Disclosure Updated Date: January 31, 2025

ASN American Society of Nephrology · JASN Journal of the American Society of Nephrology · KIDNEY360 Accessing Our World From Every Angle · CJASN Clinical Journal of the American Society of Nephrology

ASN Journal Disclosure Form

As per ASN journal policy, I have disclosed any financial relationships or commitments I have held in the past 36 months as included below. I have listed my Current Employer below to indicate there is a relationship requiring disclosure. If no relationship exists, my Current Employer is not listed.

F. Xiong has nothing to disclose.

I understand that the information above will be published within the journal article, if accepted, and that failure to comply and/or to accurately and completely report the potential financial conflicts of interest could lead to the following: 1) Prior to publication, article rejection, or 2) Post-publication, sanctions ranging from, but not limited to, issuing a correction, reporting the inaccurate information to the authors' institution, banning authors from submitting work to ASN journals for varying lengths of time, and/or retraction of the published work.

Name: Fei Xiong
Manuscript ID: K360-2024-000812R1
Manuscript Title: Update on the Assessment of Glomerular Filtration Rate in Patients with Cancer
Date of Completion: January 20, 2025
Disclosure Updated Date: January 20, 2025
