# Supplementary material for: Update on the Assessment of GFR in Patients with Cancer
Source: Kidney360. 2025 Feb 24;6(5):861–70. doi: 10.34067/KID.0000000736 (PMC12136654; doi:10.34067/KID.0000000736)
Supplement: SUPPLEMENTARY MATERIAL [file kidney360-6-861-s002.pdf]

Supplemental Material – Carboplatin Dosing According to GFR

Carboplatin is commonly and increasingly prescribed in oncology practice and is usually the drug of choice for patients who are not eligible for cisplatin.[1] In several clinical scenarios, such as non-small-cell lung cancer, extensive-stage small-cell lung cancer, and ovarian cancer, carboplatin can secure comparable efficacy with better tolerability.[2] Most importantly, carboplatin is primarily eliminated through the kidneys, with ~ 32 – 58% of the administered dose excreted unchanged in the urine with non-renal clearance due to irreversible tissue and/or protein binding).[3-5] Notably, carboplatin clearance (CaCl) depends mainly on GFR with a minor contribution of tubular secretion.[6,7] Consequently, CaCl is linearly correlated with reduced GFR which is clinically associated with a higher risk of systemic toxicity, particularly thrombocytopenia. Additionally, although less toxic than cisplatin, carboplatin is associated with acute and chronic kidney dysfunction in up to 15% of patients.[1] Thus, accurately predicting CaCl is crucial to prescribing the correct dose of carboplatin.

A landmark step in this journey occurred in 1989 when Calvert and colleagues published a formula to determine carboplatin dosing by assessing 31 patients with cancer who received 40 courses of carboplatin therapy.[3] Pharmacokinetic variables were calculated, more importantly, the area under the concentration (AUC) and total body clearance, derived from the serum level of carboplatin from multiple samples collected before, during, and up to 24 hours after the carboplatin infusion. Each patient's GFR was measured by determining the plasma clearance of ^{51}Cr-EDTA. The total body clearance of carboplatin was equal to the GFR (ml/min) (renal clearance) plus 25 ml/min (nonrenal clearance). The formula obtained was dose (mg)=target AUC ([mg/ml] xmin)x(GFR+25) (ml/min). In patients previously treated with chemotherapy, carboplatin prescribed in an AUC range of 4–6 was well tolerated (for previously untreated patients, an AUC range of 5–7). Accounting for the pharmacodynamic result of the study, a strong correlation was observed between carboplatin AUC, GFR level, and the severity of thrombocytopenia. For the first time, a reliable formula to calculate the dose of carboplatin necessary to achieve a particular AUC was available. Once mGFR was obtained and a correct AUC was set, clinicians could prescribe carboplatin safely and effectively.

After its publication, the Calvert formula was largely assessed in subsequent studies and incorporated into clinical practice.[8] It is currently recommended by reference institutions, professional societies, and clinical guidelines.[9-11] However, due to limited access to mGFR in several countries, particularly in the United States, mGFR has been substituted for GFR estimating equations over the years, more frequently the CG equation which estimates the creatinine clearance (CrCl).[8] This often leads to overestimation of carboplatin dose, and other equations have been assessed to overcome this issue, such as the MDRD and the CKD-EPI equation, with diverse and discordant results in part consequence of different methodologies assessed. Instead of mGFR, CrCl has been the most used instrument for assessing GFR, and different creatinine assays were included (e.g., Jaffe vs enzymatic, standardized vs non-standardized).[12]

More recently, it has been unequivocally demonstrated that the CG equation is associated with larger errors in the prescription of carboplatin compared with other creatinine-based models.[13,14] A landmark study from Janowitz et al. including 2,471 patients with solid tumors in England who were scheduled to receive carboplatin, assessed the error (percentage difference) in carboplatin dose based on the Calvert equation calculated using several eGFR equations comparing to the dose calculated incorporating mGFR.[13] The CG equation was observed to have the highest error in the carboplatin dose, greater than 20% in more than 25% of patients. In contrast, only 19% and 14% had errors greater than 20% with the use of CKD-EPICr and the new model (Janowitz or CamGFR equation), respectively. This study was single-center, restricted to white patients, and the serum creatinine assay was not standardized. In subsequent studies, Janowitz's group confirmed the superiority of the CKD-EPICr equation over CG, with now more than 7,000 patients assessed, incorporating multiple centers in the United Kingdom, increasing the number of Black patients and including an adjustment for the standardization of serum creatinine assay.[13,14]

Of note, the revised version of the MDRD and the CKD-EPI equations rely on standardized assays, which provide serum creatinine values that are, on average, 10%–20% lower than those of non-standardized assays used in the CG equation.[15] A few studies demonstrated that incorporation of isotope dilution mass spectrometry (IDMS) assays for creatinine on carboplatin dose relying on CG equation to estimate GFR led to

a reduction in the serum level of creatinine and an increase up to 25% on carboplatin dose. [16,17] Lawson *et al.* compared adult patients naïve to chemotherapy receiving two or more doses of the same carboplatin-based regimen (AUC of 6) in a historical series from a single center.[17] When 63 patients treated before IDMS was implemented compared to 95 patients treated after IDMS was in place, the carboplatin dose increased from 580 to 703 mg (21% average dose increase) (p<0.001). However, no increase in adverse events (grade 2 or 3 thrombocytopenia, grade 3 neutropenia, and hospitalization) during the first two cycles was observed between the two groups. Thus, the clinical impact of the creatinine assay standardization in patients receiving carboplatin remains unclear.

While the KDIGO guidelines do not specifically address the impact of different GFR equations on carboplatin clearance and dosing, a recent study helped to fulfill this gap by collecting pharmacokinetic data to demonstrate the impact of different GFR estimating equations based on creatinine and cystatin C on carboplatin dose.[18] White-Koning et al. compared the predicted versus the measured carboplatin clearance based on the drug's serum level at multiple time points in 491 patients with cancer. The Calvert equation based on CKD-EPICr-Cys was the best equation to predict CaCl, the least biased, with the lowest mean absolute percentage error (MPAE) and the lowest P20 (percentage of patients with MPAE over 20%) when compared to all other models used to predict GFR based on serum creatinine alone (CKD-EPI, CG, MDRD equations, incorporated into the Calvert or Thomas/Chatelut equations). MPAE and P20 were homogeneous in sub-groups of age, sex, and BMI. Among the formulas using only creatinine, the authors concluded that the Calvert CKD-EPI equation seems to be the most suitable creatinine-based formula to predict carboplatin clearance homogeneously in all subgroups of patients. Although the study did not assess the impact on clinical outcomes, these results reinforce the strength of the Calvert equation and agree with the KDIGO guidelines supporting the implementation of modern and validated equations, the utility of cystatin C in GFR estimation, and the advantages of mGFR or a close proxy (CaCl) in patients receiving carboplatin. Of note, although no data assessed the race-free CKD-EPI or EKFC equations in the specific context of carboplatin dosing, we agree with the KDIGO guidelines and support their use in this scenario.

The Cancer Institute of New South Wales secured the support of several international societies and gathered a panel of multidisciplinary experts to develop the International Consensus Guideline for Anticancer Drug Dosing in Kidney Disease, which has been freely available online since 2022.[11] Systematic review-based recommendations were made for a list of 54 commonly used chemotherapy drugs in addition of a chapter on GFR assessment in patients with cancer. The recommendations on carboplatin dosing include using the Calvert equation based on mGFR as the preferred methodology, particularly in the case of curative intent, extremes of body composition, and skeletal muscle waste conditions. The CKD-EPI 2009 equation (without the coefficient for race) is the upfront alternative to mGFR, expressed in ml/min, and a link for individualized dose calculation is provided.

Supplemental References

1. Gupta S, Portales-Castillo I, Daher A, Kitchlu A. Conventional Chemotherapy Nephrotoxicity. *Adv Chronic Kidney Dis*. Sep 2021;28(5):402-414.e1. doi:10.1053/j.ackd.2021.08.001
2. Pasetto LM, D'Andrea MR, Brandes AA, Rossi E, Monfardini S. The development of platinum compounds and their possible combination. *Crit Rev Oncol Hematol*. Oct 2006;60(1):59-75. doi:10.1016/j.critrevonc.2006.02.003
3. Calvert AH, Newell DR, Gumbrell LA, et al. Carboplatin dosage: prospective evaluation of a simple formula based on renal function. *J Clin Oncol*. Nov 1989;7(11):1748-56. doi:10.1200/jco.1989.7.11.1748
4. Elferink F, van der Vijgh WJ, Klein I, Vermorken JB, Gall HE, Pinedo HM. Pharmacokinetics of carboplatin after i.v. administration. *Cancer Treat Rep*. Dec 1987;71(12):1231-7.
5. Reece PA, Bishop JF, Olver IN, Stafford I, Hillcoat BL, Morstyn G. Pharmacokinetics of unchanged carboplatin (CBDCA) in patients with small cell lung carcinoma. *Cancer Chemother Pharmacol*. 1987;19(4):326-30. doi:10.1007/bf00261482
6. van der Vijgh WJF. Clinical Pharmacokinetics of Carboplatin. *Clinical Pharmacokinetics*. 1991/10/01 1991;21(4):242-261. doi:10.2165/00003088-199121040-00002
7. Van Echo DA, Egorin MJ, Whitacre MY, Olman EA, Aisner J. Phase I clinical and pharmacologic trial of carboplatin daily for 5 days. *Cancer Treat Rep*. Sep 1984;68(9):1103-14.
8. Schwenk MH. Carboplatin Dosing on the Basis of Renal Function: 30+ Years after Calvert. *Kidney360*. Feb 1 2024;5(2):271-273. doi:10.34067/kid.0000000000000349
9. BC Cancer Drug Manual - Carboplatin. BC Cancer Provincial Pharmacy. Updated February 1, 2023. Accessed September 21, 2024, 2024. http://www.bccancer.bc.ca/drug-database-site/Drug%20Index/Carboplatin_monograph.pdf
10. NCCN Chemotherapy Order Templates. National Comprehensive Cancer Network. Accessed September 21, 2024, 2024.
11. Sandhu G. AJ, Armstrong Gordon E., O'Neill N. On behalf of the ADDIKD Guideline, Group W. International consensus guideline on anticancer drug dosing in kidney dysfunction. eviQ, Cancer Institute NSW. Accessed September 21, 2024, 2024. https://www.eviq.org.au/clinical-resources/addikd-guideline/4174-anticancer-drug-dosing-in-kidney-dysfunction
12. Collins IM, Roberts-Thomson R, Faulkner D, Rischin D, Friedlander M, Mileshkin L. Carboplatin dosing in ovarian cancer: problems and pitfalls. *Int J Gynecol Cancer*. Oct 2011;21(7):1213-8. doi:10.1097/IGC.0b013e31822127ad
13. Janowitz T, Williams EH, Marshall A, et al. New Model for Estimating Glomerular Filtration Rate in Patients With Cancer. *J Clin Oncol*. Aug 20 2017;35(24):2798-2805. doi:10.1200/jco.2017.72.7578
14. Williams EH, Flint TR, Connell CM, et al. CamGFR v2: A New Model for Estimating the Glomerular Filtration Rate from Standardized or Non-standardized Creatinine in

Patients with Cancer. *Clin Cancer Res*. Mar 1 2021;27(5):1381-1390. doi:10.1158/1078-0432.Ccr-20-3201
15. Levey AS, Coresh J, Tighiouart H, Greene T, Inker LA. Measured and estimated glomerular filtration rate: current status and future directions. *Nat Rev Nephrol*. Jan 2020;16(1):51-64. doi:10.1038/s41581-019-0191-y
16. Murray B, Bates J, Buie L. Impact of a new assay for measuring serum creatinine levels on carboplatin dosing. *Am J Health Syst Pharm*. Jul 1 2012;69(13):1136-41. doi:10.2146/ajhp110560
17. Lawson J, Switchenko JM, McKibbin T, Donald Harvey R. Impact of Isotope Dilution Mass Spectrometry (IDMS) Standardization on Carboplatin Dose and Adverse Events. *Pharmacotherapy*. Jun 2016;36(6):617-22. doi:10.1002/phar.1759
18. White-Koning M, Paludetto MN, Le Louedec F, et al. Formulae recently proposed to estimate renal glomerular filtration rate improve the prediction of carboplatin clearance. *Cancer Chemother Pharmacol*. Mar 2020;85(3):585-592. doi:10.1007/s00280-019-04020-z
